# Multiple micronutrient supplementation improves micronutrient status in primary school children in Hai Phong City, Vietnam: a randomised controlled trial

**DOI:** 10.1038/s41598-021-83129-9

**Published:** 2021-02-12

**Authors:** Ngan T. D. Hoang, Liliana Orellana, Rosalind S. Gibson, Tuyen D. Le, Anthony Worsley, Andrew J. Sinclair, Nghien T. T. Hoang, Ewa A. Szymlek-Gay

**Affiliations:** 1 Institute for Physical Activity and Nutrition (IPAN), School of Exercise and Nutrition Sciences, Deakin University, Geelong, Australia; 2 Biostatistics Unit, Faculty of Health, Deakin University, Geelong, Australia; 3 Department of Human Nutrition, University of Otago, Dunedin, New Zealand; 4 National Institute of Nutrition, Hanoi, Vietnam; 5 Faculty of Health, Deakin University, Geelong, Australia; 6 Hanoi Medical University, Hanoi, Vietnam

**Keywords:** Biomarkers, Iron, Malnutrition, Anaemia, Nutrition, Nutritional supplements, Paediatric research, Obesity

## Abstract

We aimed to determine the efficacy of multiple micronutrient supplementation on the biomarkers of iron, zinc, and vitamin A status across anthropometric status categories in Vietnamese school children. In this 22-week randomised controlled trial, 347 undernourished, normal weight, or overweight/obese children aged 6–9 years were allocated to receive every school day a multiple micronutrient supplement (10 mg iron, 10 mg zinc, 400 µg vitamin A) or a placebo. Haematological indices; circulating ferritin, zinc, and retinol (corrected for inflammation); and C-reactive protein were measured at baseline and 22 weeks. At week 22, linear mixed models showed that mean corpuscular volume increased by 0.3 fL, serum ferritin by 9.1 µg/L, plasma zinc by 0.9 µmol/L, and plasma retinol by 15%, and the prevalence of zinc deficiency decreased by 17.3% points in the intervention group compared to placebo. No intervention effects were found for other haematological indices, or the prevalence of anaemia. Multiple micronutrient supplementation for 22 weeks improved the biomarkers of zinc and vitamin A status and some biomarkers of iron status, and reduced the prevalence of zinc deficiency in Vietnamese school children.

Trial registration: This trial was registered on 06/09/2016 at www.anzctr.org.au as ACTRN12616001245482.

## Introduction

The adverse effects of micronutrient deficiencies on child health are well documented and include impairments in growth, immunity, and cognitive function; reduced academic performance; and increased morbidity and mortality^1^. Although the prevalence of micronutrient deficiencies has declined worldwide over the last two decades, deficiencies of iron, zinc and vitamin A continue to be widespread and currently account for 7% of the global disease burden among school age children^2^. In Vietnam, it has been estimated that 63% of school children are at risk of zinc deficiency^3^, 6–10% are at risk of clinical vitamin A deficiency^4^, and 5–8% are at risk of iron deficiency^4^. Compared to normal weight children, micronutrient deficiencies are more prevalent in undernourished and overweight school age children^5–12^. This is concerning for Vietnam where both undernutrition and overweight are common in this population group^13^. Therefore, improving micronutrient status to combat the adverse health consequences of deficiencies in Vietnamese children has the potential to benefit those affected by both under- or over-nutrition.

The practice of single or multiple micronutrient supplementation in school children has been widespread in high-income^14,15^, as well as low- and middle-income countries including Vietnam^16–19^, and reported to reduce the prevalence of vitamin A^20^, zinc^17^ and iron deficiencies^19^, as well as anaemia^16,17,19^. However, few of these studies, and none in Vietnam, have examined their efficacy in relation to child anthropometric status^21^.

Multiple micronutrient supplementation may have a number of advantages over supplementation with a single nutrient. Firstly, the possibility of interactions among some micronutrients in multiple micronutrient supplements that may enhance the absorption, metabolism or utilisation of certain micronutrients has been recently highlighted^22–24^. For instance, zinc plays a role in the hepatic release, transport, and tissue utilisation of vitamin A via its role in the synthesis of retinol binding protein in the liver and through the action of retinol dehydrogenase. The latter is a zinc-dependent enzyme necessary for the oxidative conversion of retinol to retinaldehyde, a critical step in the visual cycle in the retina of the eye^22^. Also, supplements containing both vitamin A and iron have been shown to increase iron status to a greater extent than iron supplements alone in populations at risk of co-existing vitamin A and iron deficiencies^23^. This has been attributed to a possible role of vitamin A in the absorption of iron, as well as in the mobilisation of iron from existing spleen or liver stores into the bone marrow to support increased erythropoiesis. In some cases, however, competitive interactions between micronutrients in multiple micronutrient supplements can negatively affect their absorption and utilisation. Examples identified include the interaction between supplemental iron and zinc that may occur when given simultaneously without food^24^. Finally, the cost of the delivery of a single or multiple micronutrient supplement is likely to be the same whereas the latter is likely to have a larger health benefit^25^.

Therefore, in this study we aimed to determine the efficacy of multiple micronutrient supplementation on the biomarkers of iron, zinc, and vitamin A status of 6–9-year-old undernourished, normal weight, and overweight/obese children in rural areas of Hai Phong City, Vietnam.

## Methods

### Study design and participants

**Figure 1:**
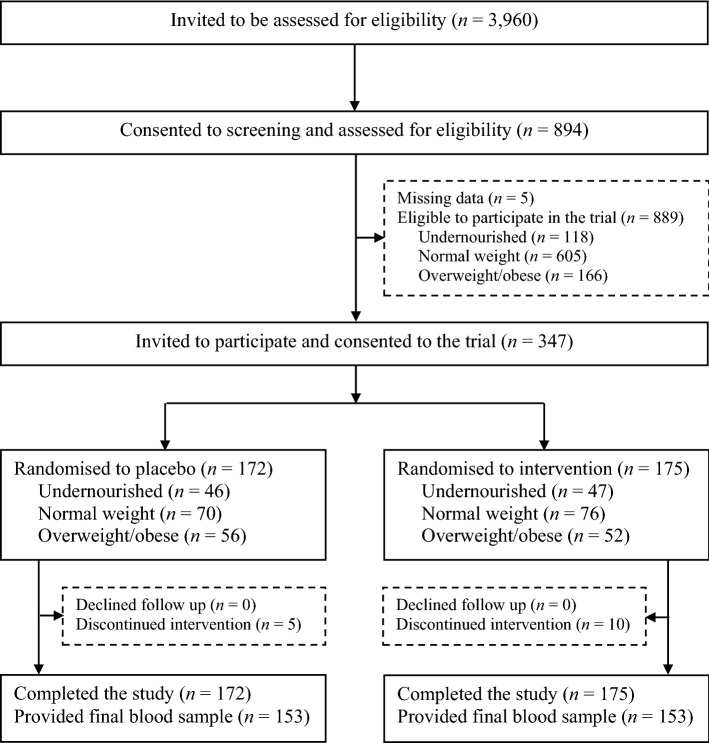
Flow of participants through the study.

This 22-week parallel, double-blind, randomised, controlled trial was conducted from December 2016 to May 2017 in rural areas of Hai Phong City, Vietnam. Eight schools were selected for participation using a multistage sampling approach described in detail elsewhere^13,26^. Briefly, two of the eight rural districts in Hai Phong City were selected at random in the first stage, followed by the selection of four schools in each district among primary schools with student enrolment of ≥ 300. All children attending grades 1–3 (ages 6–9 years; n = 3960) in the eight selected primary schools were invited to be assessed for eligibility (Fig. 1). Children were eligible to participate if they were 6–9 years old and apparently healthy. Children were excluded if they (1) had haemoglobin (Hb) concentration < 80 g/L; (2) currently consumed or planned to consume vitamin or mineral supplements; (3) had a diagnosed chronic disease or infection; (4) had a congenital abnormality; (5) had an anthropometric abnormality or (6) were severely undernourished (weight-for-age *z* score < − 3).

The trial protocol was approved by the Ethics Committee of the National Institute of Nutrition, Vietnam (610/VDD-QLKH) and the Deakin University Human Research Ethics Committee, Australia (2016-181). The trial was performed in accordance with the ethical standards laid down in the Declaration of Helsinki and its later amendments. Written informed consent was obtained from the school principal of each school and the primary caregiver of each child. In addition, verbal consent was obtained from each participating child. Participation was voluntary and participants were free to withdraw from the study at any stage. This trial was registered on 06/09/2016 at www.anzctr.org.au as ACTRN12616001245482.

### Sample size

The primary study goal was to compare a 22-week change in Hb between study groups (i.e., intervention and placebo). It was estimated that a sample size of 208 children (104 children in each group) achieved 88% power to detect a difference in a mean change of 5 g/L in Hb concentration; assuming a SD of 10 g/L^27^ at each time point and a correlation between measurement times of 0.35 (α = 0.05, two-tailed test). To account for the clustering induced by schools, the sample size was multiplied by a design effect of 1.42 [DE = 1 + ICC × (m − 1), where m = 8, the number of schools; and ICC = 0.06; the intracluster correlation coefficient]. The intracluster correlation coefficient was estimated from Hb data obtained during screening for eligibility^26^. Assuming 15% attrition rate, the target sample size for recruitment was 348 children [approximately 29 children in each strata defined by sex (boy/girl) and anthropometric status (undernourished/normal weight/overweight-obese)].

### Randomisation and concealment

Children who consented to eligibility screening and satisfied all eligibility criteria (n = 889; Fig. 1) were stratified by school, sex (boy/girl) and anthropometric status (undernourished/normal weight/overweight-obese). The list of children including only the stratification variables was provided to an independent statistician not involved in recruitment or data collection (LO). The target for recruitment was 116 children in each anthropometric status stratum. Because the number of children in these strata was not even (Fig. 1), all children in the smaller strata were invited to participate in the trial, while children in the normal weight stratum were invited following a random sequence provided by the trial statistician for each school. Invited children who consented to their participation in the trial (n = 347) were then randomly allocated to the intervention (n = 175) or placebo (n = 172) according to random sequences generated by the study statistician for each stratum (school, sex, and anthropometric status). Each child was assigned an individual study code. The codes were unblinded when data collection, biochemical analyses, and data entry were completed. Allocation concealment was achieved by secure storage of the randomisation sequence separately from the participant database.

### Intervention and placebo

All children were de-wormed with albendazole (400 mg) prior to the start of this 22-week study and again 3 months after the trial’s inception. Each morning on school days (i.e., a total of 110 school days), children were given by their teacher one capsule containing either a multiple micronutrient supplement or placebo. The supplement or placebo were consumed under teachers’ supervision with water but without food. The composition of the supplement was based on a micronutrient powder developed by the National Institute of Nutrition modified to include 10 mg zinc per capsule instead of 4.1 mg^28^. Each capsule of the multiple micronutrient supplement contained lactose, magnesium stearate, and 15 micronutrients: 10 mg iron as ferric pyrophosphate, 10 mg zinc as zinc sulphate (4.1 mg elemental zinc) and zinc gluconate (5.9 mg elemental zinc), 400 µg vitamin A, 0.5 mg vitamin B1, 0.5 mg vitamin B2, 6 mg vitamin B3, 0.5 mg vitamin B6, 0.9 µg vitamin B12, 30 mg vitamin C, 5 µg vitamin D3, 5 mg vitamin E, 150 µg folic acid, 0.56 mg copper, 90 µg iodine, and 17 µg selenium. The supplement was produced, encapsulated, tested for quality, and delivered to the schools according to the regulations of the Vietnamese Ministry of Health for food and drug administration^29^ (analysis sheet number 1693/PKN-VDD, dated 06/12/2016, issued by the National Institute of Nutrition, Vietnam). Placebo capsules contained only lactose and magnesium stearate and were produced under similar procedures as the multiple micronutrient supplement capsules. The multiple micronutrient supplement and placebo capsules were identical in terms of colour, smell, and appearance and were packaged into identical blister packs of 10 capsules. To ensure correct distribution and administration of capsules, the National Institute of Nutrition research staff not involved in recruitment or data collection labelled each blister pack with each individual child’s name, class, school, sex, and study code.

### Adherence

To assess adherence, the teachers were asked to record daily whether or not (e.g., due to school absence or refusal) children received their allocated treatment, and all used and unused blister packs were collected. These data were used to calculate the number of days on which each child received treatment and the total amount of iron, zinc, and vitamin A provided to children in the intervention group.

### Demographic, socio-economic, dietary, and anthropometric data

Demographic, socio-economic, and dietary intake data were collected at baseline. Demographic and socio-economic data were reported by the mothers via a pre-tested self-administered questionnaire. A 24-h food recall was used to collect dietary intake data by asking the mothers and children to recall all foods and beverages that the child consumed in the previous 24 h. To facilitate portion size estimation, mothers and children were shown photographs of commonly used measures (e.g., bowls, spoons) and food portion sizes (e.g., slices). Mixed dishes (e.g., spring rolls) were not disaggregated and were entered into the database as such. Energy and nutrient intakes were analysed with dietary analysis software developed by the National Institute of Nutrition in Vietnam, which accessed the Vietnamese Food Composition Tables^30^.

Weight and height were measured in duplicate at baseline using standardised methods as described in detail earlier^13^. Weight-for-age, height-for-age, and BMI-for-age *z* scores were calculated with WHO AnthroPlus^31^. Underweight was defined as weight-for-age *z* score < − 2; stunting as height-for-age *z* score < − 2; wasting as BMI-for-age *z* score < − 2; normal weight as BMI-for-age *z* score ≥ − 2 and BMI-for-age *z* score ≤ 1 in the absence of undernutrition; and overweight as BMI-for-age *z* score > 1 in the absence of undernutrition^32^. As the overweight category also included obese children, we refer to it as ‘overweight/obese’ throughout the paper. The Composite Index of Anthropometric Failure, which is an aggregate indicator of undernutrition, was used to identify undernourished children; this index defines undernutrition as either underweight, stunting, or wasting^13,26,33^.

### Blood sample collection and analysis

Non-fasting venous blood samples were collected at baseline and at the end of week 22 into a 1.5-mL EDTA-containing evacuated tube (Vacuette, Greiner Bio One, Kremsmünster, Austria) for the determination of Hb, mean corpuscular volume (MCV), haematocrit (HCT), and red blood cell distribution width (RDW-CV); a 2-mL additive-free evacuated tube (Vietlab, Vietnam) for the determination of ferritin and C-reactive protein (CRP); and a 2.5-mL heparinised trace element-free evacuated tube (Vietlab, Vietnam) for the determination of zinc and retinol. The additive-free and heparinised tubes were refrigerated immediately after collection. The heparinised tube was protected from light at all times. The time of sample collection was recorded.

Hb, MCV, HCT, and RDW-CV were determined on the day of sampling at the National Institute of Nutrition, Hanoi, Vietnam with a Micros ES 60 automated haematology analyser (HORIBA ABX, France). The measurements were validated through participation in the Randox International Quality Assessment Scheme. A pooled blood sample and certified reference material (Randox Laboratories Limited, Crumlin, UK) were analysed monthly to assess the precision and accuracy of the analytical methods. The analysed mean values for the pooled blood sample were 144.4 g/L (n = 5503) for Hb, 85.7 fL (n = 5215) for MCV, 43.2% (n = 5654) for HCT, and 15.4% (n = 3652) for RDW-CV. The analysed mean values for the quality control certified reference material were 145.8 g/L for Hb, compared to the manufacturer’s reference range of 131.7–146.7 g/L; 89.4 fL for MCV, compared to the manufacturer’s reference range of 76.3–90.3 fL; 45.1% for HCT, compared to the manufacturer’s reference range of 38.4–39.9%; and 14.9% for RDW-CV, compared to the manufacturer’s reference range of 14.9–16.6%. For Hb, the coefficient of variation was 2.1% for the pooled blood sample and 1.9% for the certified reference material. For MCV, the coefficient of variation was 5.0% for the pooled blood sample and 3.3% for the certified reference material. For HCT, the coefficient of variation was 5.8% for the pooled blood sample and 3.4% for the certified reference material. For RDW-CV, the coefficient of variation was 8.6% for the pooled blood sample and 5.1% for the certified reference material. Additionally, 10% of the study samples were analysed in duplicate for all haematological indices. The coefficients of variation for the duplicate samples were 1.7% for Hb, 2.8% for MCV, 2.3% for HCT, and 5.1% for RDW-CV.

Within one hour of sampling, the additive-free and heparinised tubes were centrifuged at 2500 rpm for 10 min at 4 °C, and the serum and plasma separated using trace-element-free techniques, and then stored immediately in an isothermal box at 4–8 °C, and frozen at − 70 °C with dry ice within 3–4 h after sampling. The serum and plasma aliquots were subsequently transported to the National Institute of Nutrition laboratory where they were stored at − 70 °C until analysis. Serum ferritin concentration was measured by ELISA using a commercial kit (Ramco Laboratories, Inc., Stafford, Texas, USA, Catalogue number S-22). Analytical accuracy was confirmed by analysis of commercially-available controls (Tri-Level Control Sera Levels 1 and 2; Ramco Laboratories, Inc., Stafford, Texas, USA). The analysed mean values for the quality control sera were 13.4 (SD 0.6) µg/L for Level 1, and 95.5 (SD 7.0) µg/L for Level 2. The certified values had a mean of 13.7 µg/L (range 8.2–19.2 µg/L) for Level 1, and 94.2 µg/L (range 60.7–127.7 µg/L) for Level 2. The inter-assay and intra-assay coefficients of variation were 4–8% and 3.2%, respectively. Serum CRP concentration was measured by an immunoturbidimetric assay using a Beckman Counter AU680 Clinical Chemistry Analyser (Beckman Counter, California, USA). Analytical accuracy for this assay was checked by analysis of commercially-available controls with certified mean values of 9.1 mg/L (range 6.3–11.8 mg/L) for Level 1, and 29.5 mg/L (range 24.1–34.9 mg/L) for Level 2 (Liquichek Immunology Control Levels 1 and 2; Bio-Rad Laboratories, Inc., California, USA). The analysed mean values for the quality control sera were 9.8 (SD 0.4) mg/L for Level 1, and 29.8 (SD 1.1) mg/L for Level 2. The inter-assay coefficient of variation was 4–7% and the intra-assay coefficient of variation was 4.0%. Plasma zinc concentration was measured according to the protocol of the International Zinc Nutrition Consultative Group^34^ with a flame atomic absorption spectrophotometer (Analytik Jena novAA 400 P, Analytik Jena AG, Germany) after dilution with 10% HNO_3_ using commercial aqueous standards (Titrisol; Merck) for external calibration. Analytical accuracy for this assay was ensured by analysis of commercially-available controls with certified mean values of 19.1 µmol/L (range 14.2–24.1 µmol/L) for Level 1, and 11.6 µmol/L (range 6.6–16.6 µmol/L) for Level 2 (Lyphochek Assayed Chemistry Control Levels 1 and 2; Bio-Rad Laboratories, Inc., California, USA). The analysed mean values for the quality control sera were 20.1 (SD 1.0) µmol/L for Level 1, and 11.3 (SD 0.9) µmol/L for Level 2. The inter-assay and intra-assay coefficients of variation were 6–9% and 7.2%, respectively. Plasma retinol was determined by reverse-phase high performance liquid chromatography (LC-10 AD VP; Shimadzu, Kyoto, Japan). All-trans retinol (purity: 98%) and retinyl acetate (purity: 95%) were used as external and internal standards (Sigma-Aldrich Corporation, Steinheim, Germany). All extraction and high performance liquid chromatography procedures were carried out under reduced light and under nitrogen in order to prevent oxidation of the compounds. The intra-assay coefficient of variation for this assay was 3.6%.

Anaemia was defined as Hb < 115 g/L^35^. Iron deficiency was defined as depleted iron stores, functional iron deficiency, or iron deficiency anaemia^35^. Depleted iron stores were defined as a serum ferritin concentration < 15 µg/L^36^ in the absence of functional iron deficiency or anaemia. Functional iron deficiency was defined as ≥ 2 abnormal values for serum ferritin (< 15 µg/L), MCV (< 80 fL)^37^, and RDW-CV (> 14.5%)^37^ in the absence of anaemia^37^. Iron deficiency anaemia was defined as Hb < 115 g/L in the presence of functional iron deficiency^37^. Low plasma zinc concentration was defined as < 9.9 µmol/L^34^. Vitamin A deficiency was defined as a plasma retinol < 0.70 µmol/L^38,39^. The prevalence of iron, zinc and vitamin A deficiencies was estimated after correcting serum ferritin, plasma zinc, and plasma retinol concentrations for inflammation for all children with a CRP concentration > 5 mg/L at a given time point (baseline and week 22), as described previously^40,41^. Plasma zinc concentrations were adjusted for time of day of blood sampling before correcting for inflammation, as described before^42^.

### Statistical analysis

Statistical analyses were conducted using an intention-to-treat approach, i.e., data were analysed by original treatment assignment, regardless of adherence. Baseline demographic, anthropometric, socio-economic, and dietary characteristics were summarised and then compared between the intervention and placebo groups using Student’s t tests for continuous variables and Chi-square tests for categorical variables.

The effect of the intervention on continuous outcomes (Hb, MCV, HCT, RDW-CV, serum ferritin, plasma zinc, and plasma retinol) was estimated using linear mixed models (LMMs) including group (intervention/placebo), time (baseline/week 22), and their interaction (group × time) as fixed effects, and school and child as random effects to account for the cluster induced by school and the repeated measures within child. We explored whether the effect of the intervention on these biomarkers was modified by child anthropometric status or sex using LMMs that included group (intervention/placebo), the effect modifier [anthropometric status (undernutrition/normal weight/overweight-obesity) or sex (boy/girl)] and their interactions (group × anthropometric status; or group × sex) as fixed effects, and school as a random effect. The outcomes for these analyses were the differences between baseline and week 22. The effect of the intervention within levels of the effect modifier is reported along with Sidak-adjusted 95% CIs. Model residuals were examined and plasma retinol concentrations were log-transformed because this improved residual normality and homoscedasticity. The models for Hb, MCV, HCT, and RDW-CV were fitted on unadjusted data, whereas the models for serum ferritin, plasma zinc, and plasma retinol concentrations used values adjusted for time of day of blood sampling (the zinc model only) and corrected for inflammation (all three models), as described above. To assess the influence of inflammation on Hb, MCV, HCT, and RDW-CV, we conducted sensitivity analyses where the same models were fitted including only children with CRP ≤ 5 mg/L; we report estimates based on all children as the conclusions of the original approach and the sensitivity analyses were similar.

Generalised LMMs with logit link and binomial distribution including study group (intervention/placebo), time (baseline/week 22) and their interaction (group x time) as fixed effects, and school and child as random effects were used to estimate the effect of the intervention on the prevalence of anaemia and zinc deficiency. Due to the low number of children with deficiencies of iron and vitamin A, we only report their overall prevalence for each time and group with no analysis.

No adjustments were made for outcome multiplicity. Statistical significance was determined by two-sided p < 0.05 in all cases. Stata (version 15.0; Stata Corp LP, TX, USA) was used for all analyses.

## Results

### Participants

None of the 347 children randomly assigned to the intervention (n = 175) or placebo (n = 172) were lost to follow up. However, 10 children in the intervention group and 5 in the placebo group discontinued their allocated treatment during the study (Fig. 1). At the completion of the study, 22 children in the intervention group and 19 in the placebo group refused to provide a blood sample.

Baseline demographic, anthropometric, socio-economic, and dietary data are shown in Table 1. The mean age across both groups at baseline was 7.2 (SD 0.8) years, 51.9% of children were boys, 64.8% of mothers were in full-time employment, and 59.6% of mothers were educated to or above high school level. The proportion of households with a monthly income of ≥ 5 million VND (i.e., at or above the average monthly household income in rural areas of Hai Phong City) was 84.0%. No statistically significant differences were found in any of the baseline characteristics presented in Table 1 between the intervention and placebo groups (all p ≥ 0.154).

**Table 1:** Demographic, anthropometric, socio-economic and dietary characteristics of children at baseline^1^.

Characteristic	Placebo (*n* = 172)	Intervention (*n* = 175)
Age, y	7.2 (0.9)	7.1 (0.8)
**Sex, n (%)**		
Girl	82 (47.7)	85 (48.6)
Boy	90 (52.3)	90 (51.4)
Weight, kg	23.4 (6.7)	22.6 (5.8)
Height, cm	119.7 (7.1)	118.9 (7.0)
Weight-for-age *z* score^2^	− 0.3 (1.7)	− 0.4 (1.6)
Height-for-age *z* score^2^	− 0.5 (1.1)	− 0.6 (1.1)
BMI-for-age *z* score^2^	− 0.0 (1.7)	− 0.1 (1.7)
**Maternal employment, n (%)**		
Self-employed	19 (15.0)	22 (17.5)
Full-time employment	84 (66.1)	80 (63.5)
Farmer	11 (8.7)	13 (10.3)
Unemployed	13 (10.2)	11 (8.7)
Missing, n	45	49
**Maternal education, n (%)**		
Below high school	55 (43.0)	48 (37.8)
High school	35 (27.3)	49 (38.6)
Above high school	38 (29.7)	30 (23.6)
Missing, n	44	48
**Monthly household income, n (%)**		
< 5 mln VND	16 (12.9)	23 (19.2)
5 to < 10 mln VND	62 (50.0)	57 (47.5)
≥ 10 mln VND	46 (37.1)	40 (33.3)
Missing, n	48	55
**Daily nutrient intake**		
Energy, kcal	1307 (311)	1309 (336)
Protein, g	50.9 (14.1)	49.4 (13.3)
Fat, g	25.6 (10.7)	26.9 (13.3)
Carbohydrate, g	219 (56)	218 (57)
Iron, mg^3^	7.3 (5.5, 9.1)	6.7 (5.5, 9.2)
Vitamin A, µg retinol activity equivalents^3^	201 (98, 357)	211 (119, 377)
Zinc, mg	6.5 (2.9)	6.3 (2.3)
Missing, n	10	3

^1^Values are mean (SD) unless otherwise indicated.

^2^*z* Scores were calculated with WHO AnthroPlus^31^.

^3^Values are median (25th, 75th percentile).

### Adherence to intervention

During the 110 school days of the 22-week study period, 151 (43.5%) children were absent from school on at least 1 day. The mean number of days of absence among these children was 3.4 (SD 3.1) days. Children absent from school did not receive the supplement or placebo capsules on those days. The mean number of capsules consumed per child during the study was 107.0 (SD 4.4) in the intervention group and 106.9 (SD 4.9) in the placebo group. The mean total amount of micronutrients received by children in the intervention group during the 22 weeks was 1071 (SD 44) mg of iron and zinc [i.e., 9.7 (SD 0.4) mg per school day], and 42,818 (SD 1749) µg of vitamin A [i.e., 389.3 (SD 15.9) µg per school day].

### Haematological and biochemical outcomes

Baseline haematological and biochemical outcomes in the intervention and placebo groups are shown in Table 2; in the subgroups of undernourished, normal weight, and overweight/obese children in Supplementary Table 1; and in girls and boys in Supplementary Table 2. We found no evidence of intervention effects on Hb, HCT, or RDW-CV (Table 2). At 22 weeks, MCV increased by 0.3 fL (95% CI 0.1, 0.5 fL), serum ferritin increased by 9.1 µg/L (95% CI 2.3, 15.9 µg/L), plasma zinc increased by 0.9 µmol/L (95% CI 0.4, 1.4 µmol/L), and plasma retinol increased by 15% (95% CI 9, 21%) in the intervention group relative to the placebo group. The exploratory analyses found no evidence that these intervention effects were modified by anthropometric status or sex (Tables 3 and 4).

**Table 2:** Means at baseline and week 22 with estimates of intervention effect for Hb, MCV, HCT, RDW-CV, serum ferritin, plasma zinc, and plasma retinol in the two study groups^1^.

	Baseline^2^		Week 22^2^		Change from baseline to week 22^3^	Intervention effect^4^	
	*n*	Mean (SE)	*n*	Mean (SE)	Mean (95% CI)	Mean (95% CI)	p-value
**Hb, g/L**							
Placebo	171	125.2 (1.9)	153	133.3 (1.9)	8.1 (4.7, 11.5)	2.4 (-2.4, 7.2)	0.333
Intervention	174	124.8 (1.9)	153	135.3 (1.9)	10.5 (7.1, 13.9)		
**MCV, fL**							
Placebo	171	82.1 (0.4)	153	81.1 (0.4)	− 1.0 (− 1.1, − 0.8)	0.3 (0.1, 0.5)	0.002
Intervention	174	82.2 (0.4)	153	81.6 (0.4)	− 0.7 (− 0.8, − 0.5)		
**HCT, %**							
Placebo	171	39.7 (0.4)	153	38.6 (0.5)	− 1.1 (− 1.9, − 0.2)	0.9 (− 0.2, 2.1)	0.115
Intervention	174	39.6 (0.4)	153	39.5 (0.5)	− 0.1 (− 0.9, 0.7)		
**RDW-CV, %**							
Placebo	171	12.1 (0.1)	153	11.9 (0.1)	− 0.2 (− 0.3, − 0.2)	0.1 (− 0.0, 0.2)	0.169
Intervention	174	12.0 (0.1)	153	11.9 (0.1)	− 0.2 (− 0.2, − 0.1)		
**Serum ferritin, µg/L**							
Placebo	168	67.1 (3.6)	152	70.0 (3.6)	2.8 (− 2.0, 7.6)	9.1 (2.3, 15.9)	0.009
Intervention	170	63.7 (3.6)	150	75.6 (3.6)	12.0 (7.1, 16.8)		
**Plasma zinc, µmol/L**							
Placebo	170	10.9 (0.4)	153	10.8 (0.4)	− 0.1 (− 0.4, 0.3)	0.9 (0.4, 1.4)	< 0.001
Intervention	173	10.5 (0.4)	151	11.3 (0.4)	0.8 (0.5, 1.2)		
**Plasma retinol, µmol/L**							
Placebo	169	1.1 (1.0)	151	1.2 (1.0)	1.05 (1.01, 1.10)	1.15 (1.09, 1.21)	< 0.001
Intervention	172	1.1 (1.0)	151	1.3 (1.0)	1.21 (1.16, 1.26)		

^1^All estimates are from linear mixed models including group (intervention/placebo), time (baseline/week 22), and their interaction (group × time) as fixed effects, and school and child as random effects. The models for Hb, MCV, HCT, and RDW-CV were fitted on unadjusted data. The models for serum ferritin, plasma zinc, and plasma retinol used values adjusted for time of day of blood sampling (the zinc model only)^42^ and corrected for inflammation (all three models)^40,41^.

*Hb* haemoglobin, *HCT* haematocrit, *MCV* mean corpuscular volume, *RDW-CV* red blood cell distribution width.

^2^Values for Hb, MCV, HCT, RDW-CV, serum ferritin, and plasma zinc are mean (SE). Values for plasma retinol are geometric mean (geometric SE).

^3^Values for all outcomes except for plasma retinol are the mean change (95% CI) within each study group between baseline and week 22. Positive values indicate within-group increases. Values for plasma retinol are a ratio of geometric means (95% CI) for baseline and week 22—all ratios are above 1 and indicate within-group increases.

^4^The intervention effect for all outcomes except for plasma retinol is the difference in the change from baseline to week 22 between the intervention group and the placebo group (95% CI)—all values are positive and indicate greater positive changes in the intervention group. The intervention effect for plasma retinol is expressed as a ratio of ratios (95% CI)—the value is above 1 and indicates a positive change in the intervention group.

**Table 3:** Mean changes within each study group between baseline and week 22 with estimates of intervention effect for Hb, MCV, HCT, and RDW-CV, by anthropometric status or sex^1^.

	Placebo^2^		Intervention^2^		Intervention effect (95% CI)^3^	p-value for interaction
	*n*	Mean (95% CI)	*n*	Mean (95% CI)		
**Hb, g/L**						
By anthropometric status^4^						0.127
Undernourished	43	5.0 (− 4.7, 14.7)	40	13.9 (4.1, 23.7)	8.9 (− 1.3, 19.2)	
Normal weight	58	6.5 (− 2.8, 15.7)	66	10.0 (0.9, 19.0)	3.5 (− 4.9, 11.9)	
Overweight/obese	52	10.1 (0.7, 19.5)	47	7.3 (− 2.2, 16.8)	− 2.9 (− 12.2, 6.5)	
By sex						0.261
Girls	68	6.0 (− 3.1, 15.1)	68	11.7 (2.6, 20.7)	5.7 (− 1.8, 13.2)	
Boys	85	8.3 (− 0.5, 17.2)	85	9.0 (0.1, 17.8)	0.6 (− 6.1, 7.3)	
**MCV, fL**						
By anthropometric status^4^						0.726
Undernourished	43	− 1.0 (− 1.3, − 0.7)	40	− 0.6 (− 0.9, − 0.3)	0.4 (− 0.1, 0.9)	
Normal weight	58	− 1.1 (− 1.4, − 0.8)	66	− 0.7 (− 1.0, − 0.5)	0.4 (− 0.0, 0.8)	
Overweight/obese	52	− 0.7 (− 1.0, − 0.4)	47	− 0.5 (− 0.8, − 0.2)	0.2 (− 0.2, 0.6)	
By sex						0.623
Girls	68	− 1.1 (− 1.4, − 0.9)	68	− 0.8 (− 1.0, − 0.5)	0.4 (0.0, 0.7)	
Boys	85	− 0.8 (− 1.0, − 0.6)	85	− 0.5 (− 0.8, − 0.3)	0.3 (− 0.0, 0.6)	
**HCT, %**						
By anthropometric status^4^						0.295
Undernourished	43	− 1.7 (− 3.7, 0.4)	40	0.5 (− 1.6, 2.6)	2.2 (− 0.4, 4.7)	
Normal weight	58	− 1.3 (− 3.2, 0.6)	66	− 0.1 (− 1.9, 1.8)	1.2 (− 0.9, 3.3)	
Overweight/obese	52	− 0.4 (− 2.4, 1.6)	47	− 0.5 (− 2.5, 1.5)	− 0.1 (− 2.5, 2.3)	
By sex						0.126
Girls	68	− 1.6 (− 3.5, 0.3)	68	0.4 (− 1.4, 2.3)	2.0 (0.1, 3.9)	
Boys	85	− 0.7 (− 2.5, 1.1)	85	− 0.4 (− 2.2, 1.3)	0.3 (− 1.4, 2.0)	
**RDW-CV, %**						
By anthropometric status^4^						0.411
Undernourished	43	− 0.2 (− 0.4, − 0.1)	40	− 0.3 (− 0.4, − 0.1)	− 0.0 (− 0.3, 0.2)	
Normal weight	58	− 0.1 (− 0.3, 0.0)	66	− 0.1 (− 0.3, 0.1)	0.0 (− 0.2, 0.3)	
Overweight/obese	52	− 0.2 (− 0.4, − 0.1)	47	− 0.1 (− 0.2, 0.1)	0.2 (− 0.1, 0.4)	
By sex						0.801
Girls	68	− 0.1 (− 0.3, 0.0)	68	− 0.1 (− 0.2, 0.1)	0.1 (− 0.1, 0.2)	
Boys	85	− 0.3 (− 0.4, − 0.1)	85	− 0.2 (− 0.3, − 0.0)	0.1 (0.1, 0.2)	

^1^All estimates are from linear mixed models including group (intervention/placebo) and anthropometric status (undernourished/normal weight/overweight-obese) or sex (boy/girl), and their interaction (group × anthropometric status; or group × sex) as fixed effects, and school as a random effect.

*Hb* haemoglobin, *HCT* haematocrit, *MCV* mean corpuscular volume, *RDW-CV* red blood cell distribution width.

^2^Values are the mean change (95% CI) within each group between baseline and week 22. Positive values indicate within-group increases.

^3^The intervention effect is the difference in the mean change from baseline to week 22 between the intervention group and the placebo group (Sidak-adjusted 95% CI). Positive values indicate greater positive changes in the intervention group.

^4^Undernourished defined as either underweight, stunting, or wasting. Underweight defined as weight-for-age *z* score < -2; stunting as height-for-age *z* score < -2; and wasting as BMI-for-age *z* score < -2. Normal weight defined as BMI-for-age *z* score ≥ -2 and BMI-for-age *z* score ≤ 1 in the absence of undernutrition. Overweight/obesity defined as BMI-for-age *z* score > 1 in the absence of undernutrition^32^.

**Table 4:** Mean changes within each study group between baseline and week 22 with estimates of intervention effect for serum ferritin, plasma zinc, and plasma retinol, by anthropometric status or sex^1^.

	Placebo^2^		Intervention^2^		Intervention effect (95% CI)^3^	p-value for interaction
	*n*	Mean (95% CI)	*n*	Mean (95% CI)		
**Serum ferritin, µg/L**						
By anthropometric status^4^						0.892
Undernourished	41	4.4 (− 5.5, 14.2)	38	16.7 (6.5, 26.9)	12.3 (− 3.9, 28.5)	
Normal weight	59	1.2 (− 7.2, 9.7)	62	10.8 (2.5, 19.1)	9.6 (− 3.5, 22.6)	
Overweight/obese	49	2.3 (− 6.9, 11.4)	47	10.3 (1.0, 19.5)	8.0 (− 6.7, 22.7)	
By sex						0.848
Girls	67	2.0 (− 5.9, 9.9)	65	12.4 (4.5, 20.4)	10.4 (− 1.3, 22.2)	
Boys	82	2.9 (− 4.4, 10.1)	82	11.9 (4.7, 19.2)	9.1 (− 1.5, 19.6)	
**Plasma zinc, µmol/L**						
By anthropometric status^4^						0.162
Undernourished	40	− 0.1 (− 0.8, 0.6)	39	1.6 (0.9, 2.3)	1.7 (0.5, 2.8)	
Normal weight	60	− 0.0 (− 0.6, 0.5)	64	0.4 (− 0.1, 1.0)	0.5 (− 0.4, 1.4)	
Overweight/obese	51	− 0.2 (− 0.8, 0.4)	47	0.6 (0.0, 1.3)	0.9 (− 0.2, 1.9)	
By sex						0.194
Girls	68	− 0.2 (− 0.8, 0.3)	65	0.3 (− 0.2, 0.8)	0.5 (− 0.3, 1.4)	
Boys	83	0.0 (− 0.5, 0.5)	85	1.2 (0.7, 1.7)	1.2 (0.4, 1.9)	
**Plasma retinol, µmol/L**						
By anthropometric status^4^						0.545
Undernourished	40	1.11 (1.01, 1.21)	39	1.21 (1.11, 1.32)	1.09 (0.96, 1.24)	
Normal weight	58	1.04 (0.96, 1.13)	63	1.19 (1.11, 1.29)	1.15 (1.04, 1.27)	
Overweight/obese	51	1.04 (0.96, 1.13)	47	1.23 (1.13 , 1.34)	1.18 (1.06, 1.32)	
By sex						0.316
Girls	66	1.02 (0.95, 1.10)	65	1.20 (1.12, 1.30)	1.18 (1.08, 1.29)	
Boys	83	1.09 (1.01, 1.17)	84	1.21 (1.13, 1.30)	1.12 (1.03, 1.21)	

^1^All estimates are from linear mixed models including group (intervention/placebo) and anthropometric status (undernourished/normal weight/overweight-obese) or sex (girl/boy), and their interaction (group × anthropometric status; or group × sex) as fixed effects, and school as a random effect. The model for plasma zinc used values adjusted for time of day of blood sampling^42^, and all models used values corrected for inflammation^40,41^.

^2^Values for serum ferritin and plasma zinc are the mean change (95% CI) within each group between baseline and week 22. Positive values indicate within-group increases. Values for plasma retinol are a ratio of geometric means (95% CI) for baseline and week 22—all ratios are above 1 and indicate within-group increases.

^3^The intervention effect for serum ferritin and plasma zinc is the difference in the mean change from baseline to week 22 between the intervention group and the placebo group (Sidak-adjusted 95% CI)—all values are positive and indicate greater positive changes in the intervention group. The intervention effect for plasma retinol is expressed as a ratio of ratios (Sidak-adjusted 95% CI)—all values are above 1 and indicate positive changes in the intervention group.

^4^Undernourished defined as either underweight, stunting, or wasting. Underweight defined as weight-for-age *z* score < − 2; stunting as height-for-age *z* score < − 2; and wasting as BMI-for-age *z* score < − 2. Normal weight defined as BMI-for-age *z* score ≥ − 2 and BMI-for-age *z* score ≤ 1 in the absence of undernutrition. Overweight/obesity defined as BMI-for-age *z* score > 1 in the absence of undernutrition^32^.

### Prevalence of micronutrient deficiencies

The raw estimates for the prevalence of anaemia and deficiencies of iron, zinc, and vitamin A are presented in Table 5. We detected no significant effect of the intervention on the prevalence of anaemia (p = 0.342). The prevalence of zinc deficiency showed a significantly larger decrease in the intervention group [change from baseline to 22 weeks: − 19.1% points (95% CI − 32.2, − 6.1% points; p = 0.004)] compared to the control group [change from baseline to 22 weeks: − 1.9% points (95% CI − 13.3, 9.6% points; p = 0.751)]; with an intervention effect of − 17.3% points (95% CI − 34.4, -0.2% points; p = 0.048).

**Table 5:** Raw estimates for the prevalence of anaemia and deficiencies of iron, zinc, and vitamin A.

	Baseline				Week 22			
	Placebo		Intervention		Placebo		Intervention	
	*n*/total *n*	%	*n*/total *n*	%	*n*/total *n*	%	*n*/total *n*	%
Anaemia^1^	22/171	12.9	29/174	16.7	8/153	5.2	9/153	5.9
**Iron deficiency**^2,3^	6/167	3.6	2/170	1.2	5/150	3.3	2/150	1.3
Depleted iron stores^4^	4/168	2.4	1/170	0.6	2/150	1.3	2/150	1.3
Functional iron deficiency^5^	1/167	0.6	0/170	0.0	3/150	2.0	0/150	0.0
Iron deficiency anemia^6^	1/167	0.6	1/170	0.6	0/150	0.0	0/150	0.0
Zinc deficiency^3,7^	65/170	38.2	77/173	44.5	56/153	36.6	45/151	29.8
Vitamin A deficiency^3,8^	14/169	8.3	8/172	4.7	3/151	2.0	0/151	0.0

^1^Defined as haemoglobin < 115 g/L^35^.

^2^Defined as depleted iron stores, functional iron deficiency, or iron deficiency anaemia^35^.

^3^Serum ferritin, plasma zinc, and plasma retinol concentrations were corrected for inflammation for all children with a C-reactive protein concentration > 5 mg/L at a given time point^40,41^.

^4^Defined as a serum ferritin concentration < 15 µg/L^36^ in the absence of functional iron deficiency or anaemia.

^5^Defined as ≥ 2 abnormal values for serum ferritin (< 15 µg/L), mean corpuscular volume (< 80 fL), and red blood cell distribution width (> 14.5%) in the absence of anaemia^37^.

^6^Defined as haemoglobin < 115 g/L in the presence of functional iron deficiency^37^.

^7^Defined as plasma zinc < 9.9 µmol/L^34^. Plasma zinc was adjusted for time of day of blood sampling before correcting for inflammation^42^.

^8^Defined as a plasma retinol < 0.70 µmol/L^38,39^.

## Discussion

This is the first trial, to our knowledge, to assess the efficacy of a multiple micronutrient supplement on the micronutrient status of school children across anthropometric status and sex categories. We found that consumption of a multiple micronutrient supplement five times a week for 22 weeks significantly increased circulating MCV, ferritin, zinc, and retinol, and reduced the prevalence of zinc deficiency in 6–9-year-old Vietnamese children in rural areas of Hai Phong City. No effects associated with the intervention were found for Hb, HCT, RDW-CV, or the prevalence of anaemia. The exploratory analyses found no evidence that the intervention effect on any of the study outcomes was modified by anthropometric status or sex. However, the study was not powered to test for modification by anthropometric status or sex.

Multiple micronutrient supplementation has been shown previously to improve plasma or serum concentrations of ferritin, retinol, and zinc, and reduce the prevalence of zinc deficiency in school children^17–19,43,44^. It was not surprising that plasma zinc concentrations increased and the prevalence of zinc deficiency reduced in the intervention group in our study because zinc deficiency was common at baseline indicating a public health problem^45^. As zinc and iron can compete for transport into enterocytes^24,46,47^, there is a concern about providing zinc and iron supplements together and without food^24,48^. It is unlikely, however, that the presence of 10 mg of iron in the supplement affected zinc absorption in our study. The iron:zinc molar ratio in the supplement we used was approximately 1:1 whereas inhibitory effect of iron supplementation on zinc absorption has only been shown when both minerals are provided together in disproportionate molar doses^24,48,49^.

Although vitamin A deficiency was rare at baseline in our study (i.e., < 10%), the multiple micronutrient supplementation effected an increase in plasma retinol concentrations. Vitamin A status has been shown previously to improve with increased intake of vitamin A not only in vitamin A-deficient school children^50^ but also in children with retinol concentrations of 0.7–1.05 µmol/L, which is indicative of marginal vitamin A deficiency^18,37,51,52^. In our study, suboptimal vitamin A status was evident in 46% of children in the intervention group at baseline, with 26% of children in this group presenting with plasma retinol concentrations of < 0.95 µmol/L. It is also possible that the observed increase in plasma retinol concentrations in our study was in part due to the increase in plasma zinc concentrations. Zinc deficiency can decrease circulating retinol concentrations even in the presence of sufficient vitamin A liver stores through its role in the hepatic synthesis and release of retinol binding protein^22^, suggesting that vitamin A status may, at least partially, depend on adequate zinc status.

Previous randomised controlled trials have found ferritin concentrations to improve as a result of increased iron intakes in school children with generally adequate iron status at baseline^53–55^. Similarly, despite the low prevalence of depleted iron stores or total iron deficiency in our study (< 3% and < 4%, respectively, in the intervention group at baseline), likely resulting from the use of iron-fortified fish sauce^56^ commonly available in Hai Phong City, we observed a significant increase in serum ferritin concentration in the intervention group. Although iron absorption is tightly regulated by iron status, a process controlled by hepcidin, with iron-sufficient individuals absorbing generally less iron compared to those affected by iron deficiency, some iron will still be absorbed and stored as ferritin if the body’s demand for iron is low^57^. We did not measure hepcidin to assess changes in body iron demand. In younger iron-replete children, concerns have been raised over the provision of supplemental iron, which has been shown to adversely affect their growth, morbidity, or gut microbial composition^58–62^, although the negative effects on growth have not been demonstrated in older children^63^. In contrast, we found no adverse effects of the intervention on child growth or morbidity^64^, although changes to gut microbial composition cannot be excluded as we did not assess it.

Except for Hb, the haematological indices we measured were generally within the accepted ranges at baseline in most children. It is therefore not surprising that we did not detect intervention effects on these indices aside from a clinically negligible change in MCV. Anaemia was common at baseline in our study (> 10%). Low Hb concentrations are often associated with iron deficiency^65^, but can also arise as a result of other micronutrient deficiencies including zinc and vitamin A^66–70^. It is unlikely that children in our study were affected by nutritional anaemias because the multiple micronutrient supplement used did not effect a change in Hb or the prevalence of anaemia despite improved circulating ferritin, zinc, or retinol concentrations. Non-nutritional factors such as genetic Hb disorders^71–73^ or parasitic infections^53^ may contribute to the burden of anaemia in this population. We were not able to determine Hb types to confirm the occurrence of genetic Hb disorders, which has been reported to range widely in Vietnam, depending on ethnicity^53,71–73^. All children were de-wormed before and in the middle of the study, which may help explain the significant increase in Hb concentration within both groups over 22 weeks despite no intervention effect.

Undernourished and overweight/obese school children are at greater risk of micronutrient deficiencies compared with normal weight children^5–12^. The micronutrient status of school age boys and girls differs as well^7,74^. The underlying reasons behind these observations vary depending on anthropometric status or sex. In undernourished children, inadequate intake of bioavailable micronutrients along with parasitic infections contribute to micronutrient deficiencies^12,75^, whereas metabolic changes apparent in obesity have been suggested to reduce micronutrient absorption or utilisation^21,76^. Differences in micronutrient status between boys and girls may be attributed to differences in requirements^77^ or intakes driven by cultural influences or gender norms^7^. Despite this, little is still known whether the efficacy of micronutrient supplementation on micronutrient status is modified by anthropometric status in school children^21^; and no research has assessed whether these intervention effects are modified by sex in this population. In iron-deficient school children, overweight/obesity appears to reduce the effect of iron supplementation on iron status^21^. Our exploratory analyses showed no evidence that the effect of the multiple micronutrient supplement was modified by anthropometric status or sex suggesting that this strategy may be useful in all Vietnamese school age children at risk of vitamin A and zinc deficiencies. This finding, however, may need further investigation in iron-deficient children, and in a larger study particularly for Hb and HCT as their effects from the intervention appeared to be slightly more beneficial for undernourished children and girls.

Strengths of our study include the randomised controlled design; excellent retention rate; high adherence to the intervention (95.7%); and the adjustment of circulating ferritin, retinol and zinc concentrations for CRP to account for the onset of inflammation using internal correction factors calculated from the study population^40,41^, although we were not able to measure α-1-acid glycoprotein and thus account for the later stages of inflammation^78^. We collected information on the time of the last meal consumed before blood sample collection as this can affect serum zinc concentration^42^. However, we deemed these data unreliable therefore we were not able to adjust plasma zinc concentrations for this variable.

In summary, multiple micronutrient supplementation 5 days a week for 22 weeks is an effective strategy for improving micronutrient status in 6–9-year-old school children in Vietnam, irrespective of anthropometric status or sex. As only poor zinc status was of public health concern and a large proportion of children presented with marginal vitamin A deficiency at baseline, the addition of iron to the multiple micronutrient supplement may not be necessary in this population, however this needs to be investigated further.

## Supplementary Information

Supplementary Information

## Data Availability

All data generated and analysed for this study are summarised in this published article. The datasets are available from the authors upon reasonable request and with permission of Deakin University and the National Institute of Nutrition in Vietnam.

## Abbreviations

CRP: C-reactive protein
Hb: Haemoglobin
HCT: Haematocrit
LMM: Linear mixed model
MCV: Mean corpuscular volume
RDW-CV: Red blood cell distribution width

## Code availability

Data described in the manuscript, code book, and analytic code will be made available to editors upon request either before or after publication for checking.
