# Supplementary material for: Multiple micronutrient supplementation improves micronutrient status in primary school children in Hai Phong City, Vietnam: a randomised controlled trial
Source: Sci Rep. 2021 Feb 12;11:3728. doi: 10.1038/s41598-021-83129-9 (PMC7881239; doi:10.1038/s41598-021-83129-9)
Supplement: Supplementary file 1 — Supplementary Information [file 41598_2021_83129_MOESM1_ESM.docx]

**Multiple micronutrient supplementation improves micronutrient status in primary school children in Hai Phong City, Vietnam: a randomised controlled trial**

**Ngan T.D. Hoang, Liliana Orellana, Rosalind S. Gibson, Tuyen D. Le, Anthony Worsley, Andrew J. Sinclair, Nghien T.T. Hoang, Ewa A. Szymlek-Gay**

**SUPPLEMENTARY TABLE 1**

Means at baseline with estimates of differences in Hb, MCV, HCT, RDW-CV, serum ferritin, plasma zinc, and plasma retinol among undernourished, normal weight and overweight/obese children^1^

|  | Undernourished^2,3^ | |  | Normal weight^2,4^ | |  | Overweight/obese^2,5^ | |  | Difference (95% CI)^6^ | Global p-value for difference |
| --- | --- | --- | --- | --- | --- | --- | --- | --- | --- | --- | --- |
|  | *n* | mean (SE) |  | *n* | mean (SE) |  | *n* | mean (SE) |  |  |  |
| Hb, *g/L* | 93 | 123.1 (1.5) |  | 144 | 123.9 (1.4) |  | 108 | 128.0 (1.5) |  |  | 0.002 |
| Undernourished vs. normal weight |  |  |  |  |  |  |  |  |  | -0.8 (-3.6, 1.9) |  |
| Undernourished vs. overweight/obese |  |  |  |  |  |  |  |  |  | -4.9 (-7.8, -1.9) |  |
| Normal weight vs. overweight/obese |  |  |  |  |  |  |  |  |  | -4.0 (-6.7, -1.4) |  |
|  |  |  |  |  |  |  |  |  |  |  |  |
| MCV, *fL* | 93 | 81.7 (0.5) |  | 144 | 82.7 (0.4) |  | 108 | 81.7 (0.5) |  |  | 0.187 |
| Undernourished vs. normal weight |  |  |  |  |  |  |  |  |  | -1.0 (-2.3, 0.3) |  |
| Undernourished vs. overweight/obese |  |  |  |  |  |  |  |  |  | -0.0 (-1.4, 1.3) |  |
| Normal weight vs. overweight/obese |  |  |  |  |  |  |  |  |  | 1.0 (-0.3, 2.2) |  |
|  |  |  |  |  |  |  |  |  |  |  |  |
| HCT, *%* | 93 | 39.1 (0.4) |  | 144 | 39.4 (0.4) |  | 108 | 40.4 (0.4) |  |  | 0.007 |
| Undernourished vs. normal weight |  |  |  |  |  |  |  |  |  | -0.3 (-1.2, 0.5) |  |
| Undernourished vs. overweight/obese |  |  |  |  |  |  |  |  |  | -1.3 (-2.2, -0.5) |  |
| Normal weight vs. overweight/obese |  |  |  |  |  |  |  |  |  | -1.0 (-1.8, -0.2) |  |
|  |  |  |  |  |  |  |  |  |  |  |  |
| RDW-CV, *%* | 93 | 12.0 (0.1) |  | 144 | 12.0 (0.1) |  | 108 | 12.1 (0.1) |  |  | 0.551 |
| Undernourished vs. normal weight |  |  |  |  |  |  |  |  |  | -0.1 (-0.2, 0.1) |  |
| Undernourished vs. overweight/obese |  |  |  |  |  |  |  |  |  | -0.1 (-0.3, 0.1) |  |
| Normal weight vs. overweight/obese |  |  |  |  |  |  |  |  |  | -0.0 (-0.2, 0.1) |  |
|  |  |  |  |  |  |  |  |  |  |  |  |
| Serum ferritin, *µg/L* | 91 | 54.5 (4.4) |  | 141 | 66.8 (4.0) |  | 106 | 73.7 (4.3) |  |  | <0.001 |
| Undernourished vs. normal weight |  |  |  |  |  |  |  |  |  | -12.3 (-20.3, -4.4) |  |
| Undernourished vs. overweight/obese |  |  |  |  |  |  |  |  |  | -19.2 (-27.6, -10.8) |  |
| Normal weight vs. overweight/obese |  |  |  |  |  |  |  |  |  | -6.9 (-14.6, 0.7) |  |
|  |  |  |  |  |  |  |  |  |  |  |  |
| Plasma zinc, *µmol/L* | 92 | 10.5 (0.4) |  | 144 | 10.8 (0.4) |  | 107 | 10.7 (0.4) |  |  | 0.501 |
| Undernourished vs. normal weight |  |  |  |  |  |  |  |  |  | -0.3 (-0.8, 0.2) |  |
| Undernourished vs. overweight/obese |  |  |  |  |  |  |  |  |  | -0.2 (-0.7, 0.3) |  |
| Normal weight vs. overweight/obese |  |  |  |  |  |  |  |  |  | 0.1 (-0.4, 0.6) |  |
|  |  |  |  |  |  |  |  |  |  |  |  |
| Plasma retinol, *µmol/L* | 91 | 1.1 (1.0) |  | 142 | 1.1 (1.0) |  | 108 | 1.2 (1.0) |  |  | 0.001 |
| Undernourished vs. normal weight |  |  |  |  |  |  |  |  |  | 0.97 (0.90, 1.05) |  |
| Undernourished vs. overweight/obese |  |  |  |  |  |  |  |  |  | 0.87 (0.90, 0.94) |  |
| Normal weight vs. overweight/obese |  |  |  |  |  |  |  |  |  | 0.90 (0.83, 0.96) |  |

^1^ All estimates are from linear mixed models including anthropometric status (undernourished/normal weight/overweight-obese) as a fixed effect and school as a random effect. The models for Hb, MCV, HCT, and RDW-CV were fitted on unadjusted data. The models for serum ferritin, plasma zinc, and plasma retinol used values adjusted for time of day of blood sampling (the zinc model only) ^1^ and corrected for inflammation (all three models) ^2,3^. Hb, haemoglobin; HCT, haematocrit; MCV, mean corpuscular volume; and RDW-CV, red blood cell distribution width.

^2^ Values for Hb, MCV, HCT, RDW-CV, serum ferritin, and plasma zinc are mean (SE). Values for plasma retinol are geometric mean (geometric SE).

^3^ Undernourished defined as either underweight, stunting, or wasting. Underweight defined as weight-for-age *z* score <-2; stunting as height-for-age *z* score <-2; and wasting as BMI-for-age *z* score <-2 ^4^.

^4^ Normal weight defined as BMI-for-age *z* score ≥-2 and BMI-for-age *z* score ≤1 in the absence of undernutrition ^4^.

^5^ Overweight/obesity defined as BMI-for-age *z* score >1 in the absence of undernutrition ^4^.

^6^ Values for all outcomes except for plasma retinol are the mean difference (95% CI) in baseline values between undernourished and normal weight children, undernourished and overweight/obese children, and normal weight and overweight/obese children. Positive values indicate greater baseline values in undernourished compared to normal weight children, undernourished compared to overweight/obese children, and normal weight compared to overweight/obese children. Values for plasma retinol are a ratio of geometric means (95% CI) for undernourished and normal weight children, undernourished and overweight/obese children, and normal weight and overweight/obese children – all ratios are below 1 and indicate lower baseline plasma retinol concentrations in undernourished compared to normal weight children, undernourished compared to overweight/obese children, and normal weight compared to overweight/obese children.

**SUPPLEMENTARY TABLE 2**

Means at baseline with estimates of differences in Hb, MCV, HCT, RDW-CV, serum ferritin, plasma zinc, and plasma retinol between girls and boys^1^

|  | Girls^2^ | |  | Boys^2^ | | Difference (95% CI)^3^ | p-value for difference |
| --- | --- | --- | --- | --- | --- | --- | --- |
|  | *n* | mean (SE) |  | *n* | mean (SE) |  |  |
| Hb, *g/L* | 165 | 125.6 (1.3) |  | 180 | 124.4 (1.2) | -1.2 (-3.5, 1.0) | 0.290 |
|  |  |  |  |  |  |  |  |
| MCV, *fL* | 165 | 82.8 (0.4) |  | 180 | 81.5 (0.4) | -1.3 (-2.3, -0.2) | 0.016 |
|  |  |  |  |  |  |  |  |
| HCT, *%* | 165 | 39.8 (0.3) |  | 180 | 39.5 (0.3) | -0.3 (-1.0, 0.3) | 0.324 |
|  |  |  |  |  |  |  |  |
| RDW-CV, *%* | 165 | 12.0 (0.1) |  | 180 | 12.1 (0.1) | 0.1 (-0.1, 0.2) | 0.290 |
|  |  |  |  |  |  |  |  |
| Serum ferritin, *µg/L* | 162 | 67.9 (3.8) |  | 176 | 63.6 (3.8) | -4.3 (-10.9, 2.2) | 0.192 |
| Plasma zinc, *µmol/L* | 164 | 10.8 (0.4) |  | 179 | 10.6 (0.4) | -0.2 (-0.6, 0.2) | 0.314 |
| Plasma retinol, *µmol/L* | 161 | 1.1 (1.0) |  | 180 | 1.1 (1.0) | 0.97 (0.91, 1.04) | 0.408 |

^1^ All estimates are from linear mixed models including sex (girl/boy) as a fixed effect and school as a random effect. The models for Hb, MCV, HCT, and RDW-CV were fitted on unadjusted data. The models for serum ferritin, plasma zinc, and plasma retinol used values adjusted for time of day of blood sampling (the zinc model only) ^1^ and corrected for inflammation (all three models) ^2,3^. Hb, haemoglobin; HCT, haematocrit; MCV, mean corpuscular volume; and RDW-CV, red blood cell distribution width.

^2^ Values for Hb, MCV, HCT, RDW-CV, serum ferritin, and plasma zinc are mean (SE). Values for plasma retinol are geometric mean (geometric SE).

^3^ Values for all outcomes except for plasma retinol are the mean difference (95% CI) in baseline values between girls and boys. Positive values indicate greater baseline values in boys compared to girls. The value for plasma retinol is a ratio of geometric means (95% CI) for girls and boys – the ratio is below 1 and indicates greater baseline plasma retinol concentrations in girls compared to boys.

**References**

1 Arsenault, J. *et al.* The time of day and the interval since previous meal are associated with plasma zinc concentrations and affect estimated risk of zinc deficiency in young children in Peru and Ecuador. *Eur J Clin Nutr* **65**, 184-190 (2011).

2 Thurnham, D. I. *et al.* Adjusting plasma ferritin concentrations to remove the effects of subclinical inflammation in the assessment of iron deficiency: a meta-analysis. *Am J Clin Nutr* **92**, 546-555 (2010).

3 Thurnham, D. I., Northrop-Clewes, C. A. & Knowles, J. The use of adjustment factors to address the impact of inflammation on vitamin A and iron status in humans. *J Nutr* **145**, 1137S-1143S (2015).

4 WHO. *Growth reference data for 5-19 years. Indicators*, <https://www.who.int/tools/growth-reference-data-for-5to19-years/indicators> (2007).
